# Double-Sided Metasurface Array for a Dual-Band and Polarization-Independent Microwave-Energy-Harvesting System

**DOI:** 10.3390/ma14216242

**Published:** 2021-10-20

**Authors:** Maged A. Aldhaeebi, Thamer S. Almoneef

**Affiliations:** 1 Department of Electronics and Communication Engineering, Hadhramout University, Mukalla P.O. Box 50512, Yemen; maged.aldhaeebi@gmail.com; 2 Electrical Engineering Department, College of Engineering, Prince Sattam Bin Abdulaziz University, Al-Kharj 11942, Saudi Arabia

**Keywords:** metasurface harvester, absorbers, energy harvesting

## Abstract

In this article, we present a simple and novel design of a double-sided metasurface for a dual-band and polarization-independent microwave-energy-harvesting system. The proposed metasurface is constructed from the dual-sided design of 8 × 8 unit cells. Different from the regular dual-band unit cells that contain two loops or multiple shapes of resonators printed in the same layer, the proposed metasurface is based on designing double loops, each combined with two arms of a dipole printed on the top and bottom sides of a single substrate. Thus, the bottom layer is utilized to generate the second frequency band of interest. Three main numerical simulations were conducted to investigate the performance of a single unit cell, a 2 × 2 supercell, and an array of an 8 × 8 metasurface structure. The numerical simulation demonstrated that 98% and 95% of the incident energy is collected at two bands of 1.8 and 6.5 GHz for the proposed harvester.

## 1. Introduction

Recent developments in the field of metamaterials opened the possibility of designing and realising near-unity harvesters, enabling many applications such as portable wireless sensor networks [1,2], RFIDs [1,3], wireless chargeable devices [1,4], the Internet of Things [5], and biomedical implantable devices [6], to name a few. An antenna and a rectification circuit are considered to be the main components to build a microwave-energy-harvesting and wireless power transfer system (MEHWS). The antenna component is utilized to receive the incident electromagnetic (EM) waves and convert them to AC power. The rectification circuit component, however, is used to convert the received AC power by the antenna part to DC [7]. The total performance of the MEHWS depends on the efficiency of each individual component combined. In order to improve the performance of the antenna part, the antenna should be effectively designed to capture an incident EM wave with different polarizations at various bands of frequencies due to the nature of the incident electromagnetic wave having an unknown polarization and frequency of operation [8,9]. Some studies enhanced the electromagnetic wave absorption performance of dual-band and single-band absorbers by using nanosheets [10,11].

Generally, a metasurface array structure has shown superior performance when compared to conventional antenna arrays, such as patch arrays, in developing an MEHWS in terms of higher harvesting efficiency [12,13,14]. Moreover, designing a metasurface harvester is different from designing an absorber where a metasurface harvester captures electromagnetic energy and dissipates it on a connected load rather than having the absorbed energy be consumed within a lossy substrate [14].

In the literature, developing a dual-band and dual-polarization receiving antenna for an MEHWS based on a metasurface antenna array has been considered a challenging task due to the need for a complicated design, which includes multiple layers and an intricate corporate feed network, used to channel the output AC and/or DC power [1,13,14,15,16,17,18,19,20,21]. Many studies concentrated on designing a dual-polarized receiving metasurface antenna array [13,16,22,23,24,25,26], and other studies focused on developing a dual-band receiving metasurface antenna array [27,28,29]. However, some studies presented dual-polarized and multiband metasurface harvesters [14,17,18,19,20,21,30,31,32,33,34]. The MEHWSs presented in the literature with dual-polarized and multiband metasurface harvesters are considered complicated and costly systems since most of them utilize a dual-layer configuration design with multiple vias connecting the stacked layers electrically. Moreover, the reported studies in [14,30,31,32,33,34] developed dual-band and dual-polarization metasurfaces using a single-layer model with multiple vias, which also add to the complexity of the overall design of the energy-harvesting system.

In this paper, the design of a metasurface array for a dual-band and polarization-independent microwave-energy-harvesting system is presented. The novel structure of the proposed metasurface array consists of a single layer and double-sided loops to achieve a dual-band and dual-polarized metasurface array with higher radiation to AC efficiency in the microwave regime. The novelty of the proposed structure is demonstrated by avoiding the use of a multilayered configuration, which requires the use of vias, which makes the design more intricate and costly. In addition, the proposed design allows for the easy integration of the rectification circuitry by placing a single diode across the joint feed of the two loops, which minimizes the overall diodes used in an array of metasurface unit cells.

## 2. A Dual-Band and Polarized Unit Cell Design Methodology

**Figure 1:**
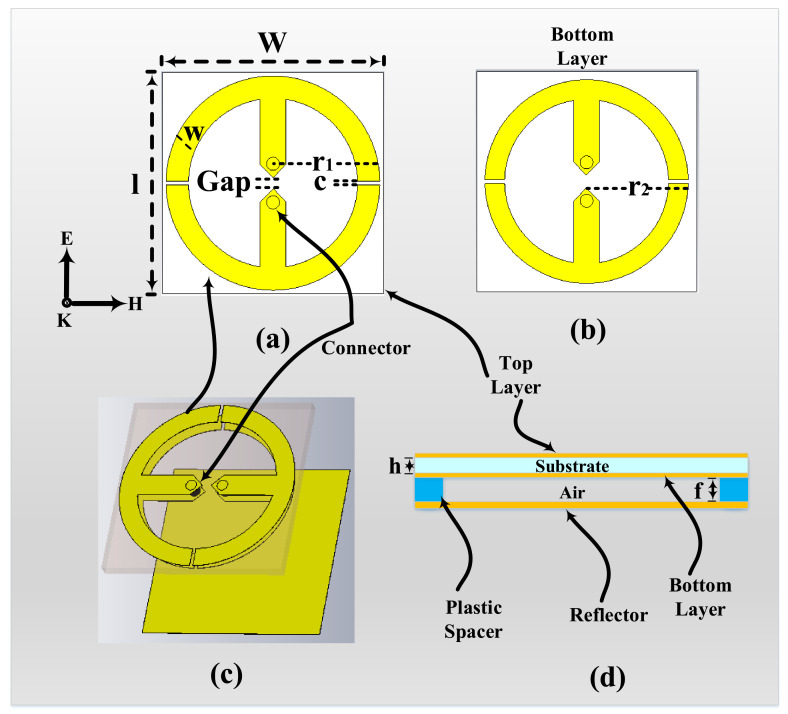
The presented unit cell: (**a**) top loop layer, (**b**) bottom loop layer, (**c**) top and bottom loop layers connected with two connectors, and (**d**) view of the top and bottom layers with the ground spacer reflector.

The design of the proposed dual-band unit cell is based on generating two bands from two loops printed on the top and bottom layers of a single Rogers 4003c substrate. The bottom layer is utilized instead of having the loops in the top layer only, thus miniaturizing the unit cell further. A single unit cell contains double-sided resonators where each side contains a loop with a dipole in the middle having a small gap. A resistive load *R* is placed on the gap of each dipole to consume the absorbed energy and to mimic a port where the rectification circuitry can be integrated, as shown in Figure 1a. The dimensions of the unit cell are w=l=23 mm with substrate thickness h=1.524 mm and copper thickness = 35 µm.

**Figure 2:**
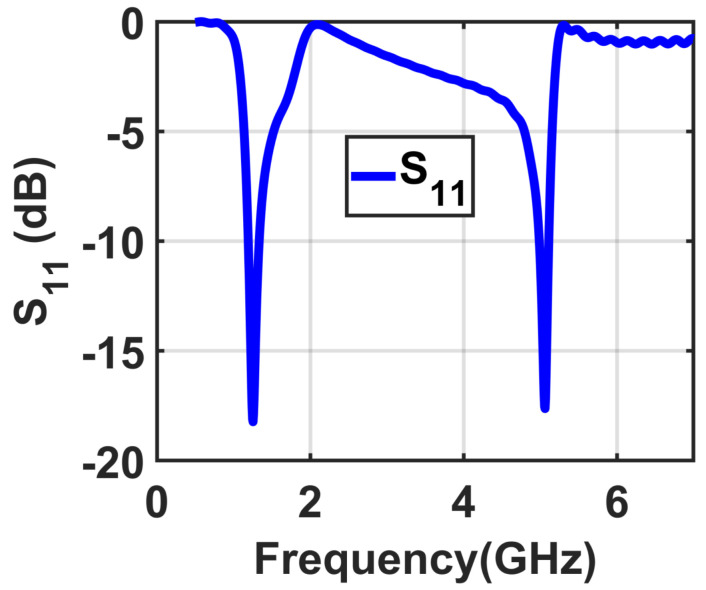
Simulation results of the proposed unit cell showing $S_{11}$.

The loop on the top layer has radius $r_{1}=11.2$ mm, whereas the radius of the bottom loop $r_{2}=10.6$ mm. The two radii were carefully studied such that the results were in two frequency bands of 1.8 GHz and 5 GHz, respectively, as shown in Figure 2. Both loops on the top and bottom layers contain two cuts *c* that divide them into two identical half loops. Each half loop on the top and bottom layer is electrically connected from the top to the bottom layer through the substrate of the unit cell, as shown in Figure 1c. In addition, a reflector is placed at a distance *f* from the bottom layer, as illustrated in Figure 1d.

In the literature, the performance of the harvester is evaluated based on a higher value of the efficiency η. This value of the efficiency η is based on two main factors including the input power $P_{in}$, which is the real power available at the surface of the harvester, and the developed power $P_{d}$, which can be calculated by summing all the load resistors of each unit cell. The efficiency η is then calculated by using the following equation: $\eta =\frac{P_{d}}{P_{in}}*100\%$ where the developed power represents all the dissipated power across the connected loads of all unit cells within the footprint. The input power is considered as the available power on the surface area or the footprint of the harvester, which can be determined by multiplying the Poynting vector by the physical area of the harvester.

Using the CST [35] simulation setup, the main design parameters of the introduced unit cell are optimized to achieve high-efficiency collected power across the resistive load. First of all, the spacer distance *f* between the bottom layer and the reflector along with the resistive R load value were investigated. The *f* values were optimized between f=5 mm and f=20 mm and the R load in between R=100Ω and R=350Ω to obtain the optimum value of *f* with the goal of achieving a higher value of radiation to AC conversion efficiency.

**Figure 3:**
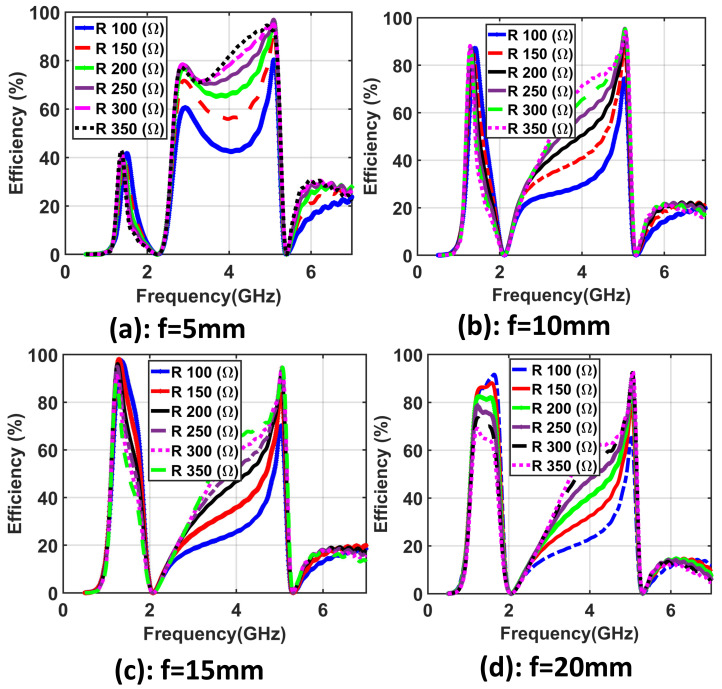
Results of the introduced unit cell demonstrating the efficiency using different values of the R load from 100 Ω to 350 Ω and using four cases of *f*: (**a**) *f* = 5 mm, (**b**) *f* = 10 mm, (**c**) *f* = 15 mm, and (**d**) *f* = 20 mm.

Simulation results demonstrating the efficiency of the unit cell with four cases f=5 mm, f=10 mm, f=15 mm, and f=20 mm while sweeping the R load values from R=100Ω to R=350Ω are shown in Figure 3a–d, respectively. The presented unit cell collected a high efficiency of 99% at the optimum values of R=200Ω and f=15 mm. Furthermore, the proposed unit cell has the capability to collect power over the entire range of R values from 100 Ω to 350 Ω, resulting in a harvester with a wide impedance bandwidth.

Next, three simulation environments were implemented to demonstrate the optimum values of other design parameters of the presented unit cell including the gap, cut, and width of the trace. First, a simulation was implemented to investigate the effect of different gap sizes. In this simulation environment, five different sizes of the gap were investigated for various values from $Gap_{1}=0.2$ mm to $Gap_{5}=0.6$ mm at optimum values of both the resistive load R of 200Ω and the distance off the spacer f=15 mm between the resonator and the reflector.

**Figure 4:**
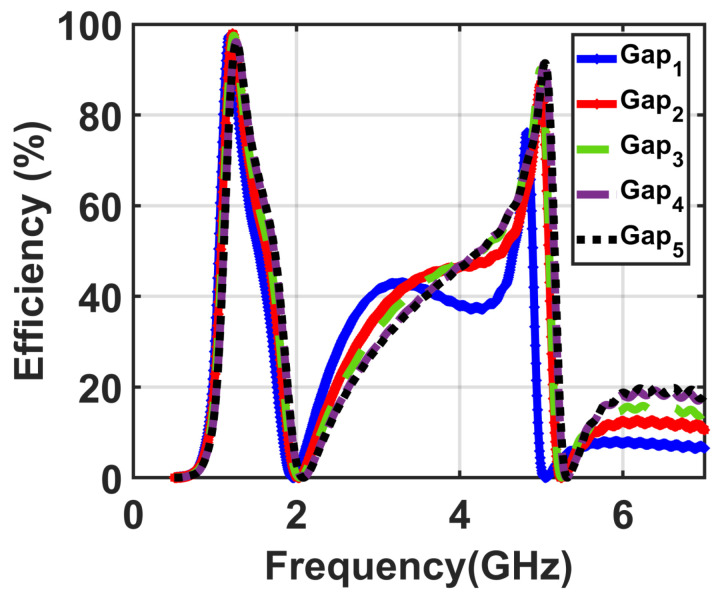
Obtained results of the efficiency of the five investigated cases for the values of the gap from $Gap_{1}=0.2$ mm to $Gap_{5}=0.6$ mm.

For all five values of the gap from $Gap_{1}=0.2$ mm to $Gap_{5}=0.6$ mm, the efficiency values were recorded to obtain the optimum value of the gap, which achieved higher efficiency, as illustrated in Figure 4. From the obtained results, we noticed that the optimum value of the gap was $Gap_{4}=0.5$ mm.

**Figure 5:**
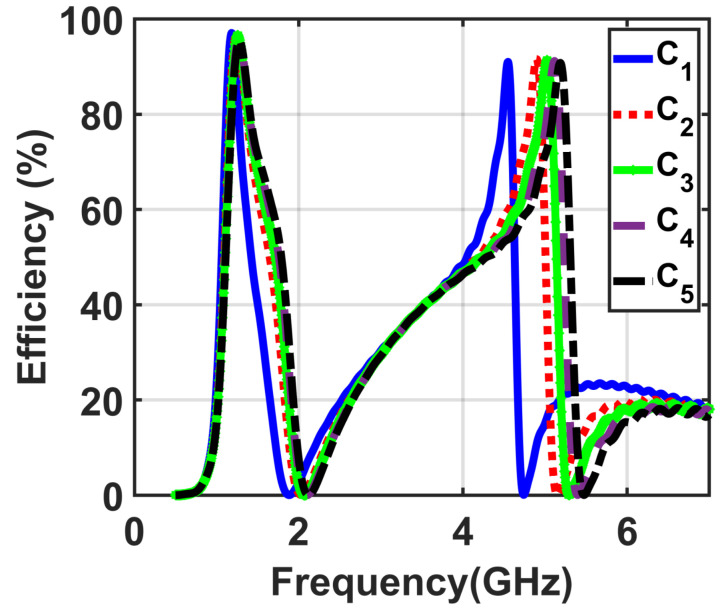
Obtained results of the efficiency of the five investigated cases for the values of the cuts from $C_{1}=0.2$ mm to $C_{5}=0.6$ mm.

The size of the cuts *c* was investigated next, while all other parameters were used at the optimal values as obtained above. To show this, a simulation environment was setup for five different values of the cuts, which changed from $C_{1}=0.2$ mm to $C_{5}=0.5$ mm with the optimal values of R=200Ω, f=15 mm, and $Gap_{4}=0.5$ mm. From the demonstrated results in Figure 5, $C_{3}=0.4$ mm is the optimum value to obtain the goal of higher efficiency.

**Figure 6:**
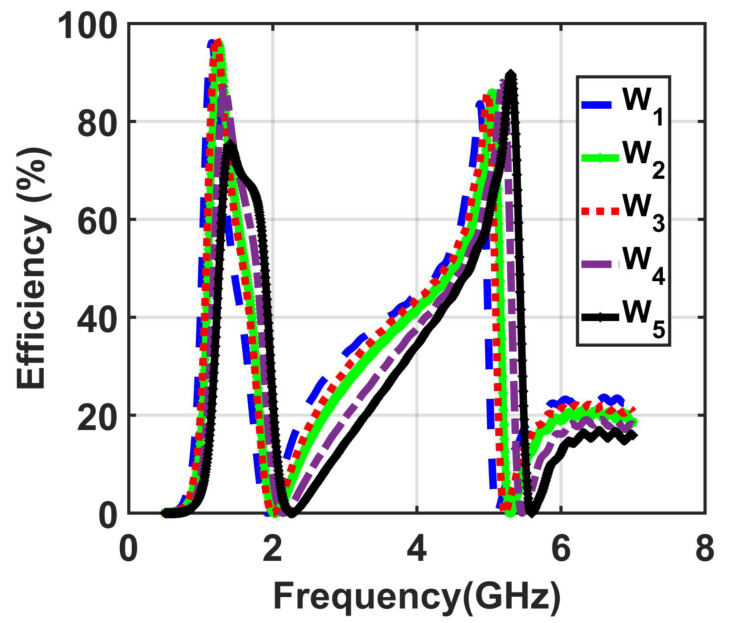
Obtained results of the efficiency of the five investigated cases for the values of the width from $W_{1}=1.5$ mm to $W_{5}=3.5$ mm.

The last numerical study was performed using five different values of the width ranging from $W_{1}=1.5$ mm to $W_{5}=3$ mm at optimal values of R=200Ω, f=15 mm, $Gap_{4}=0.5$ mm, and cut $C_{3}=0.4$ mm. Figure 6 shows that a width size of $W_{3}=2.5$ mm is the best value among the obtained results of the five investigated cases of the width from $w_{1}=1.5$ mm to $w_{5}=3.5$ mm, having the highest efficiency value.

## 3. The 2 × 2 Supercell Design Methodology

**Figure 7:**
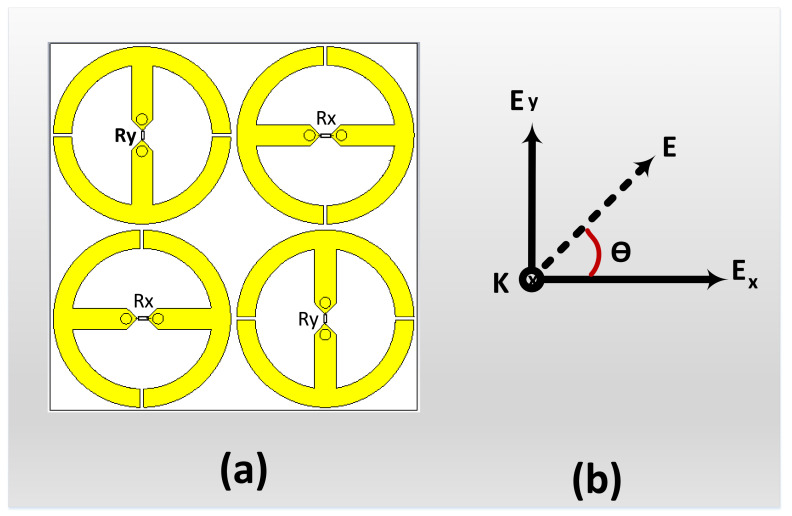
Geometry of the introduced supercell: (**a**) top loop layer and (**b**) view of the multiple polarizations at different angles of the incident power.

To demonstrate the dual-polarized feature of the presented cell, a supercell comprised of 2 × 2 unit cells was implemented. Each bottom and top layer contained four unit cells. The top left and the bottom right cells were designed for y polarizations ($R_{y}$), and the top right and bottom left cells were designed for x polarizations ($R_{x}$), as shown in Figure 7.

The design parameters of the 2 × 2 supercell including the resistive loads and the separation between adjacent cells sep were simulated and optimized to achieve high collecting efficiency.

**Figure 8:**
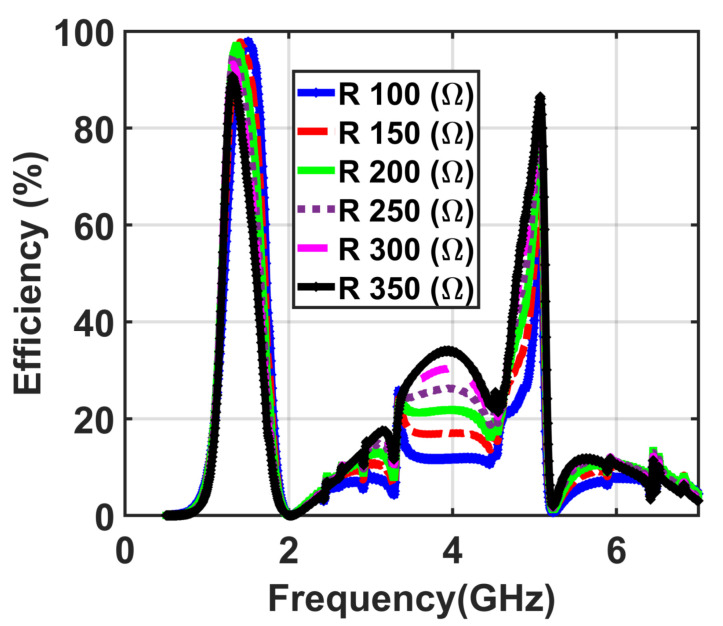
The obtained results demonstrating the efficiency of the presented supercell with different values of R ranging from 100 Ω to 350 Ω.

First, a numerical simulation was performed for different loads $R_{x}$ = $R_{y}$ values from 100Ω to 350Ω at the optimum parameters obtained for the single unit cell. Figure 8 shows the numerical efficiency results of the proposed dual-band and dual-polarized 2 × 2 supercell with different values of the terminated loads ranging from 100 Ω to 350 Ω.

**Figure 9:**
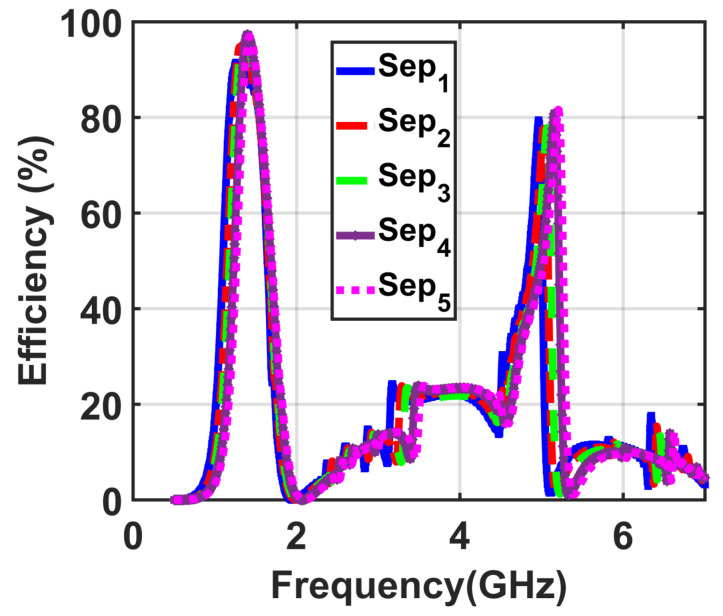
Obtained results demonstrating the efficiency with the separation between adjacent cells of the implemented supercell varied.

The next simulation was implemented to optimize the second design parameter of the proposed supercell including the separation between adjacent cells sep. The values of sep were optimized with the goal of having higher conversion efficiency. Figure 9 shows the efficiency results of the presented supercell with different values of sep ranging from $Sep_{1}=0.2$ mm to $Sep_{5}=0.6$ mm. The results showed that the optimum value of $Sep_{4}$ achieves the highest conversion efficiency when the length is 0.4 mm.

**Figure 10:**
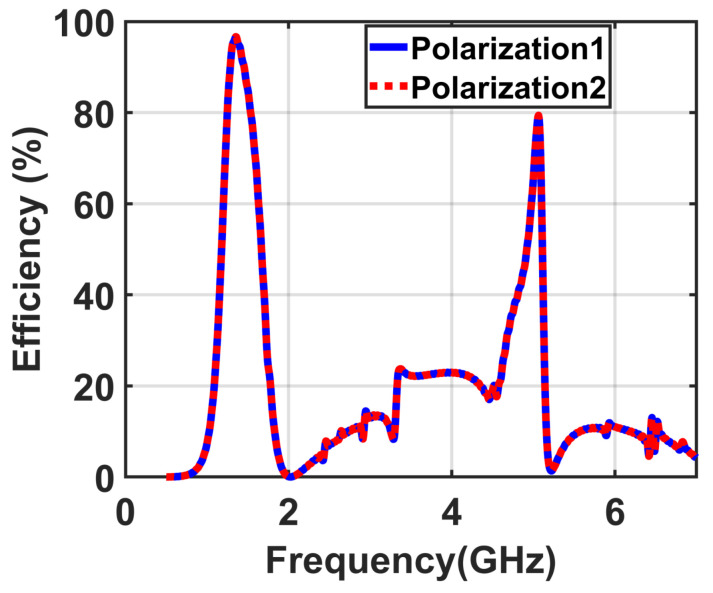
Obtained results illustrating the efficiency of the implemented supercell for both TE and TM modes.

To study the capability of the supercell to absorb electromagnetic energy with dual-polarization, a simulation was conducted for two normal incidence TE and TM mode polarizations. At the two bands of 1.9 GHz and 6.5 GHz, the efficiency is 98% and 80% for both TE and TM mode polarizations, respectively, as illustrated in Figure 10.

**Figure 11:**
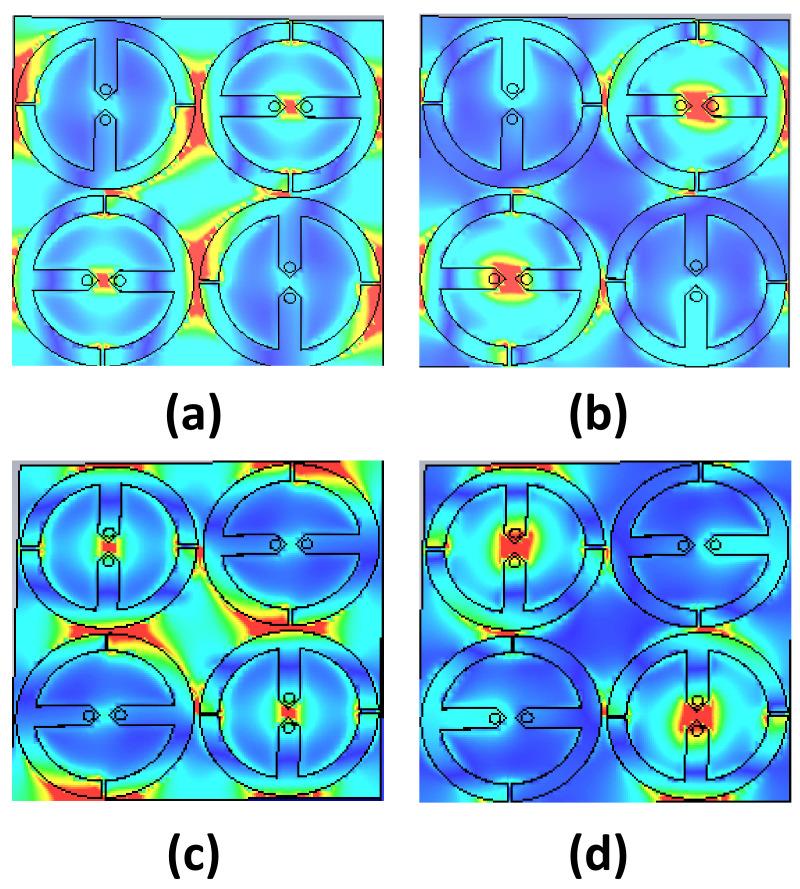
Obtained results of the implemented supercell illustrating the magnitude of the E-field across the surface of the proposed super unit cell by the red colour for (**a**) TE mode (Polarization 1) at 1.9 GHz, (**b**) TE mode (Polarization 1) at 5 GHz, (**c**) TM mode (Polarization 2) at 1.9 GHz, and (**d**) TM mode (Polarization 2) at 5 GHz.

To illustrate the duality of the polarization of the implemented supercell for both bands, a simulation environment was implemented for both TE and TM polarizations. Figure 11 illustrates the obtained results of the electric field across the surface of the supercell for two polarizations, where the red colour demonstrates the high-magnitude values of the electric field for both TE and TM modes.

## 4. The 8 × 8 Array Metasurface Design Methodology

**Figure 12:**
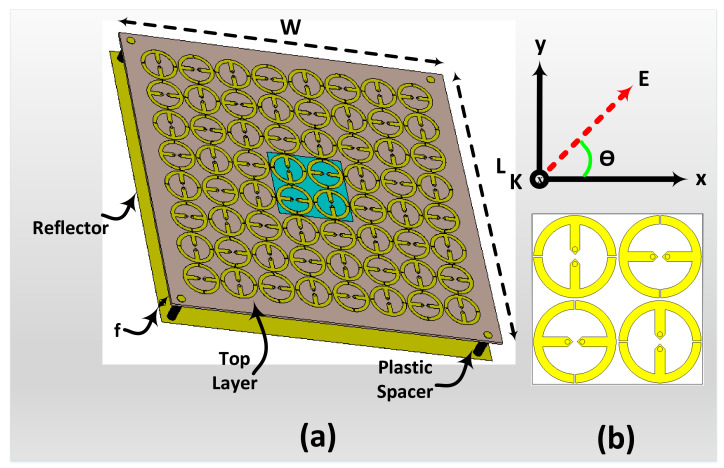
(**a**) Geometry of the implemented 8 × 8 metasurface, (**b**) available power from different incident angles.

The proposed metasurface harvester comprised 8 × 8 identical unit cells, as shown in Figure 12. The overall array size of the periodic array structure is 200 mm × W=200 mm. In the simulation environment setup, each unit cell in the proposed harvester was terminated by the optimum R of 200Ω.

In the simulation environment, the presented metasurface was simulated as a harvester in receiving mode to collect the power from different angles of incidence using a plane wave excitation, as demonstrated in Figure 12b.

**Figure 13:**
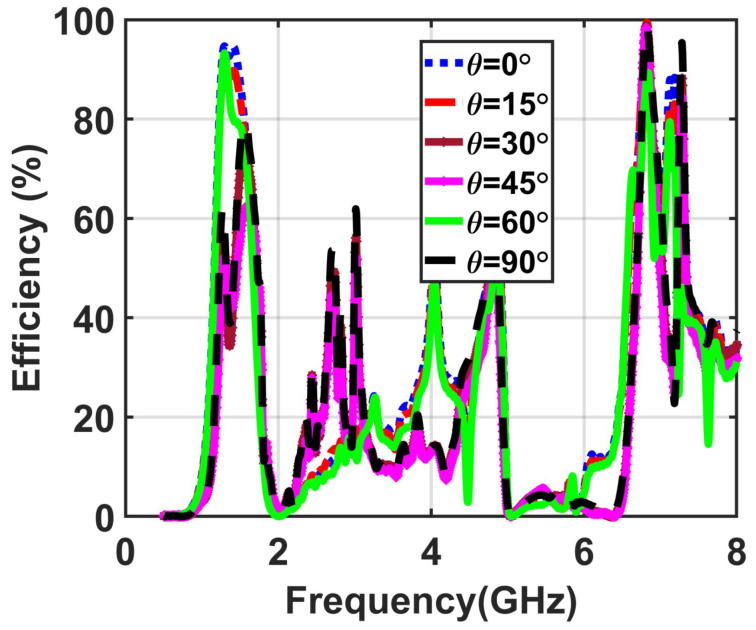
Obtained results demonstrating the efficiency with various incident angles of the implemented 8 × 8 metasurface.

In the simulation setup, the incident wave was used with different values of the incident angle (θ from 0${}^{\circ }$ to 90 ${}^{\circ }$) to study the ability of the proposed metasurface to collect the available power from different incident angles, as shown in Figure 12b. Figure 13 demonstrates the efficiency results of the proposed metasurface for various incident angles (θ).

The results showed that for all the incident angles of the plane wave, the efficiency of the metasurface array is 98% and 95% at both bands of operating frequencies. Figure 13 shows the overlapping curves of the efficiencies, indicating the ability of the introduced metasurface to harvest the incident electromagnetic waves from different angles of incidence equally.

To demonstrate the novelty of the proposed dual-band and polarization-independent metasurface, Table 1 shows the advantages of the proposed metasurface array presented in this paper compared with a number of state-of-the-art dual-band and dual-polarized arrays that are based on metasurfaces presented in the literature. The proposed metasurface design allows for a simple connection of tightly placed unit cells where integrating a matching network has two main advantages compared with other developed metasurface arrays presented in the literature. The first feature of the proposed metasurface is the wideband matching impedance. Such an impedance bandwidth is defined as the range of impedances that results in a harvesting efficiency of 75% or higher. From the results presented in Figure 3, a wide range of impedances from 50 Ω to 350 Ω resulted in efficiencies of at least 75%. Another advantage of the proposed metasurface is that such a wideband impedance response allows for the possibility of integrating a wide range of diodes without the need for a matching network between the diode and the electromagnetic collector.

## 5. Conclusions

The novel design of a planar, simple, multipolarized, and dual-band metasurface was presented. The proposed metasurface was constructed from 8 × 8 unit cells, which provide high efficiencies at various incident angles with various terminated load values. The novelty of the presented metasurface for energy harvesting was demonstrated through several numerical studies, which showed the ability of the proposed metasurface to achieve a higher efficiency of 98% and 95% for two operating frequency of 1.8 and 6.5 GHz with a multipolarized incident wave.

## Figures and Tables

**Table 1:** A comparative study of the proposed dual-band and polarization-independent metasurface array with various state-of-the-art published papers.

Reference	Frequency Bands (GHz)	Unit Cell Size	Bandwidth of Matching Impedance	Need for Matching Network	Efficiency %
[17]	5.5 and 7.2	0.22λ	Narrowband	Yes	94 and 93%
[19]	2.45 and 6	0.23λ	Narrowband	Yes	90 and 85%
[30]	1.85 and 2.45	0.32λ	Narrowband	Yes	47and 23 %
[31]	2.7 and 5	0.31λ	Narrowband	Yes	91 and 84 %
[32]	1.68 and 2.12	0.67λ	Narrowband	Yes	67 and 36 %
This work	1.8 and 6.5	0.25λ	Wideband	NO	98 and 95%

## Data Availability

The data presented in this study are available on request from the corresponding author.
